# BRCA1-IRIS inactivation overcomes paclitaxel resistance in triple negative breast cancers

**DOI:** 10.1186/s13058-014-0512-9

**Published:** 2015-01-13

**Authors:** Zannel Blanchard, Bibbin T Paul, Barbara Craft, Wael M ElShamy

**Affiliations:** Cancer Institute, University of Mississippi Medical Center, 2500 N. State Street, Jackson, MS 39216 USA; Department of Medicine, University of Mississippi Medical Center, 2500 N. State Street, Jackson, MS 39216 USA; Present address: University of Connecticut Health Center, 263 Farmington Avenue, Farmington, CT 06030 USA

## Abstract

**Introduction:**

Intrinsic or acquired chemoresistance is a major problem in oncology. Although highly responsive to chemotherapies such as paclitaxel, most triple negative breast cancer (TNBC) patients develop chemoresistance. Here we investigate the role of BRCA1-IRIS as a novel treatment target for TNBCs and their paclitaxel-resistant recurrences.

**Methods:**

We analyzed the response of BRCA1-IRIS overexpressing normal mammary cells or established TNBC cells silenced from BRCA1-IRIS to paclitaxel *in vitro* and *in vivo*. We analyzed BRCA1-IRIS downstream signaling pathways in relation to paclitaxel treatment. We also analyzed a large cohort of breast tumor samples for BRCA1-IRIS, Forkhead box class O3a (FOXO3a) and survivin expression. Finally, we analyzed the effect of BRCA1-IRIS silencing or inactivation on TNBCs formation, maintenance and response to paclitaxel in an orthotopic model.

**Results:**

We show that low concentrations of paclitaxel triggers BRCA1-IRIS expression *in vitro* and *in vivo*, and that BRCA1-IRIS activates two autocrine signaling loops (epidermal growth factor (EGF)/EGF receptor 1 (EGFR)-EGF receptor 2 (ErbB2) and neurogulin 1 (NRG1)/ErbB2-EGF receptor 3 (ErbB3), which enhances protein kinase B (AKT) and thus survivin expression/activation through promoting FOXO3a degradation. This signaling pathway is intact in TNBCs endogenously overexpressing BRCA1-IRIS. These events trigger the intrinsic and acquired paclitaxel resistance phenotype known for BRCA1-IRIS-overexpressing TNBCs. Inactivating BRCA1-IRIS signaling using a novel inhibitory mimetic peptide inactivates these autocrine loops, AKT and survivin activity/expression, in part by restoring FOXO3a expression, and sensitizes TNBC cells to low paclitaxel concentrations *in vitro* and *in vivo*. Finally, we show BRCA1-IRIS and survivin overexpression is correlated with lack of FOXO3a expression in a large cohort of primary tumor samples, and that BRCA1-IRIS overexpression-induced signature is associated with decreased disease free survival in heavily treated estrogen receptor alpha-negative patients.

**Conclusions:**

In addition to driving TNBC tumor formation, BRCA1-IRIS overexpression drives their intrinsic and acquired paclitaxel resistance, partly by activating autocrine signaling loops EGF/EGFR-ErbB2 and NRG1/ErbB2-ErbB3. These loops activate AKT, causing FOXO3a degradation and survivin overexpression. Taken together, this underscores the need for BRCA1-IRIS-specific therapy and strongly suggests that BRCA1-IRIS and/or signaling loops activated by it could be rational therapeutic targets for advanced TNBCs.

## Introduction

Paclitaxel is a powerful chemotherapy for many cancers, such as breast, prostate and ovarian cancers [1-5], as well as chemotherapy-refractory cancers such as small cell lung cancer [6-8]. Paclitaxel polymerizes tubulin to disrupt normal microtubule dynamics leading to cell death [4]. Despite preclinical and clinical success, intrinsic or acquired paclitaxel resistance remain a challenge in oncology [9-12].

Survivin, a structurally unique member of the inhibitor of apoptosis proteins family (IAP) is involved in cell division and apoptosis [13-15]. Survivin is a poor prognostic factor in several tumor types, and is involved in tumor cell resistance to ionizing radiation and chemotherapies for example, paclitaxel [16-20]. In fact, survivin expression is induced following paclitaxel exposure in breast cancer cells [21,22]. Survivin expression is negatively controlled by the Forkhead box class O (FOXO) transcription activity [23,24] and therefore is positively controlled by activated protein kinase B (AKT) [24-27].

FOXO proteins play a pivotal role in the regulation of cell cycle arrest, cell death and protection from stress stimuli [23]. Perturbation of FOXO’s function deregulates cell proliferation and leads to accumulation of DNA damage [23,25]. AKT, or mitogen-activated protein kinases (MAPK), phosphorylate FOXOs at specific sites, causing its nuclear exclusion and degradation [23,26]. Constitutive AKT activation is frequently correlated with cytoplasmic Forkhead box O3a (FOXO3a) and decreased patient survival in breast cancer and other malignancies [25,27,28]. Drugs like paclitaxel achieve their therapeutic effects through activation of FOXO3a [29-31].

Triple negative breast cancer (TNBC) is an aggressive breast cancer subtype lacking estrogen receptor (ER) and progesterone receptor (PR) expression and human epidermal growth factor receptor 2 (HER2) gene amplification. Hence, patients with this subtype lack targeted therapies. TNBC constitutes approximately 20% of all breast cancers in the USA and is overrepresented in young African American women. TNBC tumors have the poorest prognosis and tend to grow and spread to other parts faster than other cancers and often harbor BRCA1 mutations or lack of expression. Although, initially responsive to paclitaxel, TNBCs often recur with chemotherapy-resistant, visceral and brain metastasis.

The *BRCA1* locus product, BRCA1-IRIS, shares 1,365 residues with the full-length product of this locus, the tumor suppressor, BRCA1 [32,33]. Despite that, BRCA1-IRIS is a genuine oncogene in breast and ovarian cancers. Indeed, BRCA1-IRIS overexpression induces over-replication [32], over-proliferation by upregulating Cyclin D1 expression [34,35], apoptosis-resistance in human mammary (HME) and ovarian surface (HOSE) epithelial cells by inactivating p53 and/or activating AKT/survivin [36,37]. The majority of breast tumors, especially TNBCs express high levels of BRCA1-IRIS associated with increased p-AKT and survivin expression, and lack of BRCA1 expression [38]. Interestingly, BRCA1-IRIS-overexpressing HME cells when injected in SCID mice mammary fat pads develop invasive TNBCs that also show increased AKT and survivin expression and/or activation and lack BRCA1 expression [38].

Understanding the various mechanisms leading to paclitaxel resistance may help in the design of novel, more accurate therapies [12]. Here, we show BRCA1-IRIS overexpression is involved in TNBCs intrinsic and acquired paclitaxel resistance, through, in part, increasing expression and activation of autocrine signaling loops involving epidermal growth factor receptor 1 (EGFR) and epidermal growth factor receptor 3 (ErbB3) that activate AKT leading to FOXO3a degradation and survivin overexpression. BRCA1-IRIS inactivation using a novel inhibitory mimetic peptide reversed these effects and significantly reduced TNBC cells growth, survival and aggressiveness, *in vitro* and *in vivo*. More importantly, this peptide sensitized established preclinical TNBCs to low paclitaxel concentrations. BRCA1-IRIS inactivation represents a novel and attractive target for TNBCs.

## Methods

### Cell culture

Generation and maintenance of the immortalized HME cells and its variants, BRCA1-IRIS-overexpressing (HME/IRIS) cell lines, were described earlier [32,36]. In brief, immortalized HME cells were generated from mammary epithelial cells purified from tissues isolated from mammary gland reductions (Clontech Laboratories, Mountain View, CA, USA) using standard techniques by a suitable human telomerase reverse transcriptase (TERT)-expressing virus and selection [32]. Full-length BRCA1-IRIS cDNA was cloned into the pRevTRE plasmid (retrovirus version, Clontech Laboratories) to produce the pRevTRE-IRIS retrovirus. Retroviral particles were generated by transfecting 293T cells with pRevTRE-IRIS together with all necessary packaging plasmids. On days 2 and 3 viral supernatant was collected, pooled, and used as is to infect immortalized HME cells stably expressing p-TetOn plasmids (Clontech Laboratories, with suitable selection). Infected cells were then selected using hygromycine for 2 weeks, and 10 to 15 clones were generated, all referred to HME/IRIS. Ectopic BRCA1-IRIS expression in these clones is induced by exposure to 2 μg/ml of doxycycline (Invitrogen, Carlsbad, CA, USA). BRCA1-IRIS was verified at the beginning and periodically using western blot using mouse anti-human BRCA1-IRIS-specific antibody. While variation between clones exists, in every clone the expression is approximately two- to fivefold above the level in normal immortalized HME cells and resembling that found endogenously expressed in TNBC cell lines. Other cell lines, such as MDA-MB-231 (HTB-26), MDA-MB-468 (HTB-132) and BT-549 (HTB-122) cell lines were from American Type Culture Collection (ATCC) and were maintained in RPMI 1640 medium containing 10% fetal bovine serum (FBS). All cell lines used in this study were transfected with a retrovirus expressing the luciferase gene.

### Antibodies

Rb anti-survivin (#2808), −EGF (ab9695), −EGFR (ab23430), −p-ErbB2 (ab131104), −ErbB3 (ab20161), −FOXO3a (ab47409), −FOXO1 (ab39670), and -p-FOXO (T32, ab26649), or m anti-p-EGFR (Y1173; ab24912), −H2B (ab52484) and -PCNA (ab18197) were from Abcam (Cambridge, MA, USA). Rb anti-ErbB2 (#2165), −Cyclin D1 (#2978), −PTEN (#9188), −AKT (#2938), −p-AKT (S463, #4060), −ERK (#4695), −p-ERK (T202/Y204, #4370), −JNK (#9258), −p-JNK (T183/Y185, #9255), −p38 (#8690), −p-p38 (T180/Y182, #2387), −Bcl2 (#2870), −Bcl-xL (#2762) and -Skp2 (#4358) were from Cell Signaling Technology (Danvers, MA, USA). The m anti-NRG1 (MAB377) was from R&D Systems (Minneapolis, MN, USA). The m anti-nuclear factor kappa B (NF-κB)/p65 (IMG-150A) was from Imgenex (San Diego, CA, USA), the Rb anti-MDM2 (s1357) was from Epitomics (Burlingame, CA, USA) and the m anti-actin (cp01) was from Calbiochem (San Diego, CA, USA). Mouse monoclonal anti-human anti-BRCA1-IRIS was developed in our laboratory.

### Small interfering RNA transfections, and small hairpin RNA construction and generation of stable knockdown cell lines

Small interfering RNA (siRNA) transfections and protocol were described previously [32]. BRCA1-IRIS small hairpin RNA (shRNA) was designed using the ‘shRNA Design Tool’ from the IDT website [39], (sequence available upon request), inserted between BamHI and EcoR1 sites in pSIREN-RetroQ plasmid (Addgene, Cambridge, MA, USA).

### Immunohistochemistry (IHC) staining and scoring

Breast tissue microarrays comprised of normal, ductal carcinoma *in situ* (DCIS), invasive and metastatic samples were purchased from US Biomax, Inc. (Rockville, MD, USA). IHC protocols were described earlier [38]. A semi-quantitative scoring system was used to identify the percentage of tumor cells showing positive staining [40]. Scoring represents: overall stain intensity and percentage of cancer cells stained in four high magnification fields for each sample. Average overall staining intensity [41] was valued as percentage of cell stained/field: zero (<1% staining) was considered negative; 1 (1 to 10% staining) was considered weakly stained; 2 (10% to 50% staining) was considered medium stained and 3 (>50% staining) was considered strongly stained. The positive staining scoring method is totally subjective and artifacts such as high background or variable stain deposition can skew the results and the scores for the two categories remain as separate functions and cannot be combined for analysis and comparison [42].

### *In vivo* tumorigenicity assay

All animal experiments were approved by the Institutional Animal Care and Use Committee (IACUC) of the University of Mississippi Medical Center. SCID (Jackson Laboratory, Bar Harbor, ME, USA) or Nu/Nu (Harlan Laboratories, Indianapolis, IN, USA) female mice were used. Protocols were previously described [38].

### BRCA1-IRIS inhibitory peptide

A synthetic peptide corresponding to amino acids 1365–1399 of BRCA1-IRIS protein (see [32] for sequence) conjugated to cell and nuclear penetrating sequence was used.

### Cell viability measurement

Cell viability under different experimental conditions was determined using cell counting or MTS assay.

### Cell migration assay

μ-Dish (35mm, high Culture-Inserts, ibidi GmbH, Munich, Germany) was used. Inserts surrounded control or BRCA1-IRIS shRNA MDA-MB-231 or MDA-MB-468-expressing cells until confluence. At which time, inserts were removed, floating cells washed and attached cells allowed to migrate for 24 h. A montage of multiple pictures representing the whole well was mounted digitally together and migration calculated from a fixed point. Each experiment was done in triplicate repeated three separate times.

### Cell invasion assay

Growth factor-reduced BD matrigel™ invasion chambers (24-well plate, 8.0μm, BD BioCoat™) were used (BD Biosciences, San Jose, CA, USA). Invaded cells were Crystal Violet stained 7 days later, photographed and counted. Each experiment was done in triplicate repeated three separate times.

### Mammosphere assay

Ultra-low attachment 6-well plates (Corning Life Sciences, Union City, CA, USA) were used. Every third day, medium was exchanged with one containing treatments for up to 10 days when mammospheres were counted and photographed. Each experiment was done in triplicate repeated three separate times.

### *In vivo* efficacy of BRCA1-IRIS inhibitory peptide

Female Nu/Nu mice (6 to 8 weeks old) were injected with 2 x 10^6^ of MDA-MB-468 cells in the second right and fourth left mammary gland. Mice bearing tumors of approximately 100 mm^3^ were randomly grouped to receive DMSO (intraperitoneally (i.p.)) + scrambled peptide (10 mg/kg) intratumorally (i.t.), IRIS peptide (10 mg/kg, i.t.), paclitaxel (10 mg/kg, i.p.), or IRIS peptide (5 mg/kg, i.t.) + Taxol (5 mg/kg, i.p.) every third day for four times per experiment. Tumor volume was measured by caliper and is represented as percentage of volume at day 0 of treatment. At the end point, tumors or their remnants were collected, fixed in 10% formalin and histologically or immunohistochemically analyzed.

## Results

### BRCA-IRIS overexpression triggers paclitaxel resistance, *in vitro*

We recently showed BRCA1-IRIS overexpression promotes intrinsic as well as acquired cisplatin resistance in ovarian tumor and HOSE cells, respectively [37]. We also showed that BRCA1-IRIS silencing or inactivation using a novel inhibitory peptide reversed both [37]. Thus, we hypothesized that BRCA1-IRIS overexpression could also be involved in paclitaxel resistance developed in TNBC patients [4,9].

**Figure 1:**
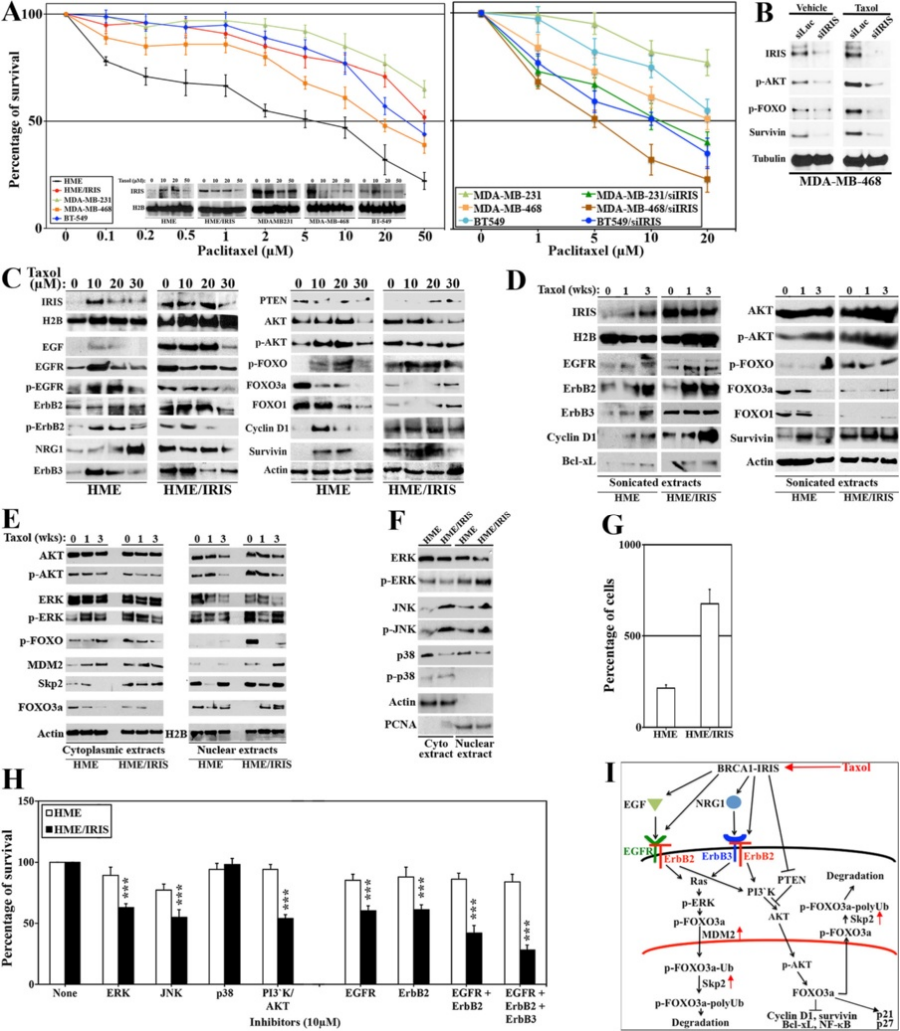
**BRCA1-IRIS overexpression promotes intrinsic and acquired paclitaxel resistance in TNBC cells. (A)** The survival of HME, HME/IRIS and the indicated TNBC cell lines following treatment with increasing concentrations of paclitaxel. Values are means of triplicates done three separate times. Inset shows BRCA1-IRIS expression in these cell lines following exposure to increasing concentrations of paclitaxel. **(B)** The expression of the indicated proteins following treatment with vehicle or paclitaxel (5 μM) for 24 h or MDA-MB-468 previously silenced from luciferase or BRCA1-IRIS for 48 h. **(C)** The expression of the indicated proteins in HME or HME/IRIS cells following exposure to 0, 10, 20 or 30 μM of paclitaxel. **(D)** The expression of the indicated proteins in HME or HME/IRIS cells following exposure to 1 μM of paclitaxel for 0, 1 or 3 weeks. **(E** and **F)** The expression of the indicated proteins in the nucleus or cytoplasm of HME or HME/IRIS following exposure to 1 μM of paclitaxel for 0, 1 or 3 weeks. Activated ERK, JNK and p38 were detected using antibodies specifically detect p-^T202/Y204^-ERK, p-^T183/Y185^-JNK, and p-^T180/Y182^-p38. **(G)** The effect of BRCA1-IRIS overexpression on the proliferation of HME cells. **(H)** The effect of inhibiting ERK (using PD98059), JNK (using SP600125), p38 (using SB203580), PI3′K/AKT (using LY294002), EGFR (using Erlotinib), ErbB2 (using CP-724714), EGFR/ErbB2 (using Lapatinib), and EGFR/ErbB2/ErbB3 (using Sapitinib) on the survival of HME or HME/IRIS cells. Values represent the means of experiments that were performed in triplicate done three separate times, ^***^ = *P* ≤0.001 (compared to control in each cell line)*.* **(I)** Schematic representation of the data so far. EGFR, ErbB2 and ErbB3, epidermal growth factor receptor 1, 2 and 3, respectively; HME, human mammary epithelial cells; TNBC, triple negative breast cancer.

To study BRCA1-IRIS overexpression on the intrinsic or acquired paclitaxel resistance, HME/IRIS and three TNBC cell lines; MDA-MB-231, MDA-MB-468 and BT549 or HME cells were used. All cell lines were exposed to increasing concentrations of paclitaxel and cells viability and BRCA1-IRIS expression were measured. HME cells were the most sensitive cell line, showing an IC_50_ of approximately 5 μM (black line in Figure 1A, left). MDA-MB-468 cells were the most sensitive TNBC cell line [43], showing an IC_50_ of approximately 20 μM (orange line in Figure 1A, left), whereas BT549 and MDA-MB-231 were more resistant showing IC_50_ of approximately 35 μM (blue line in Figure 1A, left) and >50 μM (green line in Figure 1A, left), respectively. Interestingly, HME/IRIS cells were as resistant as MDA-MB-231 cells showing an IC_50_ of approximately 50 μM (red line in Figure 1A, left). Moreover, resistance level was well correlated with BRCA1-IRIS expression in each cell line. Cell lines showing high basal level of BRCA1-IRIS and maintaining a high level after treatment, such as BT549, HME/IRIS and MDA-MB-231, were more resistant than those that had high basal of BRCA1-IRIS but failed to maintain a high level after treatment, such as MDA-MB-468 (see inset in Figure 1A, left). Taken together, these data suggest a direct relationship between the level of BRCA1-IRIS and the intrinsic paclitaxel resistance in TNBC cells.

### Direct role for BRCA1-IRIS overexpression in TNBC cells paclitaxel resistance

To show the direct role of BRCA1-IRIS overexpression in TNBC cells paclitaxel intrinsic resistance, MDA-MB-231, MDA-MB-468 and BT549 cells were transfected with luciferase (siLuc, negative control) or BRCA1-IRIS (siIRIS)-specific siRNAs for 48 h before they were exposed to increasing concentration of paclitaxel and survival was measured by cell counting 24 h later. Alternatively, silenced cells were treated with 5 μM of paclitaxel and proteins isolated 24 h later were interrogated by western blot analysis.

Compared to siLuc-transfected cells, BRCA1-IRIS-silenced cells survival was significantly decreased when treated with paclitaxel. Indeed, in the more resistant TNBC cell lines, BT-459 or MDA-MB231, IC_50_ dropped from ≥50 μM (light blue and green line, respectively Figure 1A, right) to approximately 10 μM (dark blue and green line, respectively Figure 1A, right) when BRCA1-IRIS was silenced in them. Moreover, even the more sensitive MDA-MB-468 cells became more sensitized and the IC_50_ dropped from approximately 20 μM (light-brown line, Figure 1A, right) to 5 μM when BRCA1-IRIS was silenced in them (dark-brown line, Figure 1A, right).

On the molecular level, compared to vehicle-treated cells, paclitaxel treatment induced BRCA1-IRIS, p-AKT, p-FOXO, survivin and Cyclin D1 in MDA-MB-468 cells (compare siLuc on the right to the left in Figure 1B). In the absence of BRCA1-IRIS, this induction as well as the basal level of these survival factors was significantly decreased (compare siIRIS on the right and the left in Figure 1B). Taken together, these data clearly show that some if not all paclitaxel-intrinsic resistance in TNBC cells depends on BRCA1-IRIS overexpression.

### Signaling loops involved in BRCA1-IRIS-induced intrinsic and acquired paclitaxel resistance

Although, HME cells were the least resistant to paclitaxel (Figure 1A, left), BRCA1-IRIS expression increased in them following paclitaxel treatment, especially at lower concentrations (inset in Figure 1A, left). This made us wonder whether *in vivo* too naïve HME cells exposed to low paclitaxel concentrations respond by upregulating BRCA1-IRIS expression in order to survive. Now genetically altered and BRCA1-IRIS overexpressing, these cells could pose an imminent risk later if they survived and grow as a paclitaxel-resistant TNBC clone.

Thus, to define on the molecular level the role of BRCA1-IRIS in paclitaxel-acquired and intrinsic resistance, we analyzed HME and HME/IRIS cells exposed to 0, 10, 20 or 30 μM of paclitaxel for 24 h. Paclitaxel-acquired increase in BRCA1-IRIS, EGFR and ErbB3 (not epidermal growth factor receptor 2 (ErbB2)), epidermal growth factor (EGF) and neurogulin 1 (NRG1) expression in HME cells was documented (Figure 1C, left). All were intrinsically high in HME/IRIS and remained unchanged after treatment (Figure 1C, left). In keeping with that, a paclitaxel-acquired increase in EGFR activation detected as an increase in p-Y^1173^-EGFR, which is induced by EGF binding to the EGFR-ErbB2 complex and ErbB2 activation detected as increase in p-Y^1248^-ErbB2, which is induced by NRG1 binding to the ErbB2-ErbB3 complex in HME cells, were detected (Figure 1C, left). Both were intrinsically high in HME/IRIS cells and remained unchanged after treatment (Figure 1C, left).

Furthermore, a paclitaxel-acquired decrease in PTEN level in HME cells, while intrinsically low in HME/IRIS cells, remained unchanged after drug treatment was documented (Figure 1C, right). Concurrently, a paclitaxel-acquired increase in AKT phosphorylation on S^308^/T^473^ and FOXO3a phosphorylation on T^32^ (AKT target) in HME cells, while intrinsically high p-AKT and p-FOXO levels in HME/IRIS cells remained unchanged after treatment were documented (Figure 1C, right). In keeping with that, a paclitaxel-acquired decrease in total FOXO3a and FOXO1 levels in HME cells, while already low and remained so in HME/IRIS cells following paclitaxel treatment were observed (Figure 1C, right). In line with that, a paclitaxel-acquired increase in FOXO targets; for example, Cyclin D1 and survivin (also known BRCA1-IRIS targets, see [34-37]) in HME, whereas intrinsically high levels remained unchanged in HME/IRIS cells were detected (Figure 1C, right).

To ascertain that even further, we generated paclitaxel-resistant HME and HME/IRIS cells by exposing them to 10 μM of paclitaxel for 1 or 3 weeks (cells were then propagated in paclitaxel-free media). Consistent with the data described above, these paclitaxel-resistant HME cells also showed a paclitaxel-acquired increase in BRCA1-IRIS, EGFR, ErbB2, ErbB3, p-AKT, p-FOXO3a, concurrent with decrease in total FOXO3a and FOXO1 and increase in Cyclin D1, survivin and Bcl-xL in HME (Figure 1D). All were intrinsic in HME/IRIS cells and remained unchanged after drug treatment (Figure 1D). Taken together, these data suggest that intrinsic or paclitaxel-acquired resistance in TNBC cells is due to high expression level of BRCA1-IRIS.

### BRCA1-IRIS overexpression promotes FOXO3a degradation

Phosphorylation by AKT and/or ERK promotes FOXO3a nuclear exclusion and degradation [28]. Total AKT levels were not changed in the cytoplasm or the nucleus of paclitaxel-resistant HME or HME/IRIS cells (Figure 1E). In contrast, the level of p-AKT was slightly higher in the cytoplasm, while slightly lower in the nucleus of paclitaxel-resistant HME cells than HME/IRIS cells (Figure 1E). While similar in the cytoplasm, total ERK decreased in the nucleus of paclitaxel-resistant HME and HME/IRIS cells (Figure 1E). The level of p-ERK increased in the cytoplasm of paclitaxel-resistant HME cells, decreased in the cytoplasm of paclitaxel-resistant HME/IRIS cells, while remained largely unchanged in the nucleus of resistant HME and HME/IRIS cells (Figure 1E). The level of p-FOXO increased in the cytoplasm of paclitaxel-resistant HME cells, while it decreased in the cytoplasm of resistant HME/IRIS cells (Figure 1E). No p-FOXO in the nucleus of paclitaxel-resistant HME cells, while relatively high level in paclitaxel-resistant HME/IRIS cells was detected (Figure 1E). MDM2 and Skp2 have been previously implicated in mono- and poly-ubiquitylation, respectively of FOXO proteins. We detected increased level of MDM2 and Skp2 in the cytoplasm and nucleus of the paclitaxel-resistant HME and HME/IRIS (Figure 1E). Taken together, these data suggest that intrinsic or paclitaxel-acquired increase in MDM2 and Skp2 expression induced by BRCA1-IRIS overexpression cooperatively promote FOXO3a ubiquitylation and proteasomal degradation in HME/IRIS and HME cells, respectively.

### Additional signaling pathways induced by BRCA1-IRIS overexpression

We also observed that compared to HME, HME/IRIS cells contained lower ERK and p38 while higher JNK levels in the cytoplasm and nucleus (Figure 1F). In contrast, compared to HME cells, HME/IRIS cells contained lower level of p-ERK in the cytoplasm but higher level in the nucleus (Figure 1F), higher levels of p-JNK in the cytoplasm and nucleus (Figure 1F), and similar level of p-p38 in the cytoplasm but no p-p38 in the nucleus in both cell lines (Figure 1F). Taken together, these data suggest additional signaling pathways induced by BRCA1-IRIS overexpression [34-36] that could also be involved in promoting FOXO3a degradation.

### Selective sensitivity of BRCA1-IRIS-overexpressing cells to drugs against these pathways

The above data imply BRCA1-IRIS-overexpressing cells sensitivity to inactivation of these pathways. To evaluate that, similar numbers of HME or HME/IRIS cells were grown in the presence of 10 μM of ERK (PD98059), JNK (SP600125), p38 (SB203580), PI3′K/AKT (LY294002), EGFR (Erlotinib), ErbB2 (CP-724714), EGFR/ ErbB2 (Lapatinib) or EGFR/ErbB2/ErbB3 (Sapitnib) inhibitors for 24 h.

First, because BRCA1-IRIS overexpression significantly increased proliferation of HME cells (Figure 1G and see [34-36]) the data obtained with the inhibitors were normalized to untreated cells of each cell line, separately. None of the drugs at the concentration used had an effect on HME cells survival (white bars in Figure 1H). In contrast, ERK, JNK, PI3′K/AKT, EGFR and ErbB2 inhibitors decreased HME/IRIS cells survival by approximately 50% compared to untreated HME/IRIS cells (black bars in Figure 1H). More dramatic effect was noticed using the dual EGFR/ErbB2 inhibitor and even more dramatic using the inhibitor that targets EGFR, ErbB2 and ErbB3 at the same time (Figure 1H). Taken together, these data suggest that BRCA1-IRIS-overexpressing cells are more sensitive to drugs that block EGFR-ErbB2 and ErbB2-ErbB3 complexes and their downstream signaling pathways. Schematic representation of all the above data is presented in Figure 1I.

### A BRCA1-IRIS mimetic inhibitory peptide abolishes BRCA1-IRIS expression and functions

**Figure 2:**
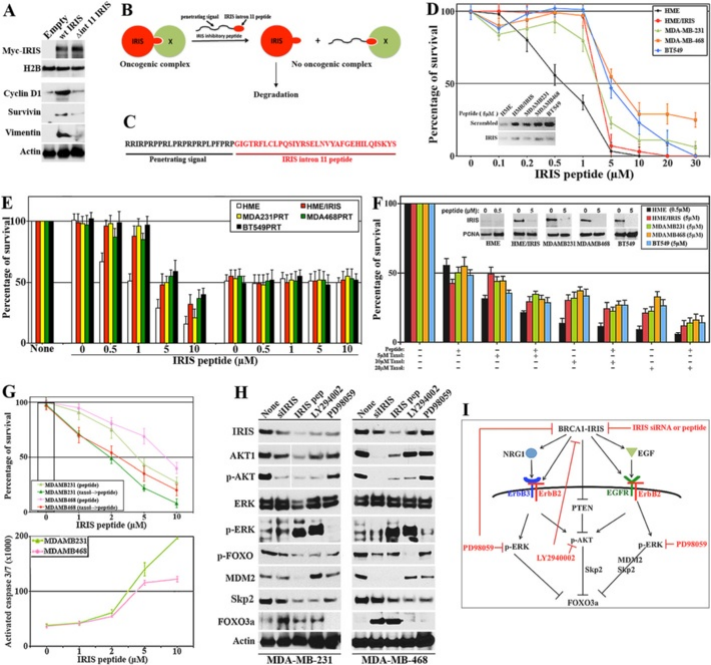
**BRCA1-IRIS inhibitory peptide effect on TNBC cells survival,** ***in vitro*** **. (A)** Expression of indicated proteins in HME cells transfected with empty vector, Myc-tagged wild-type BRCA1-IRIS (wt IRIS) or Myc-tagged mutant BRCA1-IRIS (missing the intron 11 domain, Δint 11 IRIS) for 48 h. **(B)** Schematic representation of the proposed function of BRCA1-IRIS intron 11 domain and peptide. **(C)** BRCA1-IRIS inhibitory peptide; black sequence is penetrating signal and red sequence is BRCA1-IRIS intron 11 domain. **(D)** Survival of the indicated cells exposed to increasing concentrations of IRIS peptide. Values are means of triplicates done three separate times. Inset: effect of 5 μM of scrambled or IRIS peptide on BRCA1-IRIS expression in the indicated cell lines. **(E)** Effect of increasing concentrations of IRIS peptide on the indicated cell lines following luciferase or BRCA1-IRIS silencing. Values are means of triplicates done three separate times. **(F)** The synergistic effect between IRIS peptide (5 μM) in the indicated TNBC cell lines and 0, 5, 10 and 20 μM of paclitaxel. Values are means of triplicates done three separate times. Inset: effect of 0.5 μM (HME) or 5 μM (other cell lines) of IRIS peptide on BRCA1-IRIS expression. **(G**, upper**)** Effect of paclitaxel at 1 μM alone (white bar), increasing concentrations of IRIS peptide alone (light-colored lines) or the combination (dark-colored lines) on the survival of indicated cells. Values are means of triplicates done three separate times. **(G**, lower**)** The gradual increase in activated caspase 3/7 in the indicated cells following exposure to increasing concentrations of IRIS peptide. Values are means of triplicates done three separate times. **(H)** The expression of the indicated proteins in MDA-MB-231 and MDA-MB-468 cells following transfection of BRCA1-IRIS siRNA, or the exposure to 5 μM IRIS peptide, 10 μM of PI3′K/AKT inhibitor (LY294002) or ERK1/2 inhibitor (PD98059). **(I)** Schematic representation of the data in Figure 2. HME, human mammary epithelial cells; TNBC, triple negative breast cancer.

The above data seem to argue that inactivating BRCA1-IRIS could sensitize TNBC cells to paclitaxel. Since BRCA1-IRIS-specific inhibitor is not yet available, the following reasons motivated us to pursue the inhibitory mimetic peptide approach instead. First, previous results suggested that BRCA1-IRIS connect with partners using a domain in its C-terminus [32,34]. Second, unlike full-length BRCA1-IRIS, overexpression of an intronless BRCA1-IRIS (Δint11, missing the 34 amino acid domain encoded by intron 11) failed to induce expression of targets such as; Cyclin D1, survivin and vimentin in HME cells (see Figure 2A and [34-38,44]). Based on these facts, we hypothesized that BRCA1-IRIS forms an oncogenic complex(es) by connecting with other factor(s) (Figure 2B) and that transduction of BRCA1-IRIS intron 11 domain could in a dominant-negative fashion disrupt this interaction leading to loss of BRCA1-IRIS oncogenic effect (Figure 2B).

To test this hypothesis, we synthesized a peptide (hereafter IRIS peptide) consisting of the BRCA1-IRIS intron 11 peptide (red sequence in Figure 2C) fused to a cell/nuclear penetrating signal at the N-terminus (black sequence in Figure 2C). Equal numbers of HME, HME/IRIS, MDA-MB-231, MDA-MB-468 and BT549 cells were exposed to increasing concentrations of scrambled or IRIS peptides for 48 h. Compared to scrambled peptide, treatment with IRIS peptide decreased survival in all cell lines. HME cells showed an IC_50_ of approximately 0.5 μM, whereas HME/IRIS and the TNBC cell lines an IC_50_ of 5 to 7.5 μM (Figure 2D). On the molecular level, treatment with 5 μM of IRIS peptide for 24 h almost completely abolished BRCA1-IRIS expression in all these cell lines compared to treatment with 5 μM scrambled peptide (inset in Figure 2D). Taken together, these data support our hypothesis that IRIS peptide acts as a dominant negative in TNBC cells suppressing BRCA1-IRIS oncognecity by destabilization of the protein (Figure 2B).

To ascertain the specificity of IRIS peptide even further, HME, HME/IRIS, MDA-MB-231, MDA-MB-468 and BT549 cells were first transfected with siLuc or siIRIS for 48 h before exposing them to increasing concentrations of IRIS peptide for another 48 h. Gradual decrease in all cell lines was detected in siLuc-transfected cells following treatment with increasing concentrations of IRIS peptide (Figure 2E, left). In contrast, BRCA1-IRIS silencing significantly decreased survival of all cell lines (Figure 2E, right) and treatment with IRIS peptide had no additive effect on these cells even at higher concentrations (Figure 2E, right). These data, suggest that IRIS peptide targets BRCA1-IRIS specifically leading to significant growth retardation and cell death in TNBC cells, *in vitro*.

### BRCA1-IRIS inhibition sensitizes breast cancer cells to low paclitaxel concentrations, *in vitro*

Whether inactivating BRCA1-IRIS could sensitize TNBC cells to paclitaxel was investigated next. HME cells exposed to 0.5 μM or HME/IRIS, MDA-MB-231, MDA-MB-468 and BT549 cells exposed to 5 μM of IRIS peptide were exposed or not to 5, 10 or 20 μM of paclitaxel for an additional 24 h. At these concentrations, IRIS peptide completely abolished BRCA1-IRIS expression from all cell lines (Figure 2F, inset) and reduced survival by approximately 50% in all cell lines (Figure 2F). While paclitaxel also decreased survival of all cell lines that gradually increased with increasing the concentration, the combination had even more pronounced effect on the survival of all cell lines (Figure 2F).

To identify the lowest paclitaxel concentration that could synergize with IRIS peptide, MDA-MB-231 and MDA-MB468 cells were treated with 1 μM of paclitaxel, increasing concentrations of IRIS peptide or both for 24 h. As shown above, survival gradually decreased with increasing IRIS peptide concentration in both cell lines (light-colored lines in Figure 2G, upper), which correlated very well with gradual increase in activated caspase 3 and 7 in both cell lines (Figure 2G, lower). Although at this very low concentration of paclitaxel we detected no cell death in either cell line (white bar in Figure 2G, upper), addition of 1 μM additively increased the effect of IRIS peptide on the survival of both cell lines (dark-colored lines in Figure 2G, upper). Taken together, these data suggest synergy between BRCA1-IRIS inhibition and very low concentrations of paclitaxel on the survival of TNBC cells, *in vitro*.

### Molecular comparison between the effect of BRCA1-IRIS silencing or inactivation in TNBC cells

To further explore IRIS peptide mechanism of action on cell survival and at the same time generate further evidence for its specificity, MDA-MB-231 and MDA-MB-468 cells were silenced from BRCA1-IRIS for 72 h, treated with 5 μM of IRIS peptide for 24 h and for comparison cells were exposed to 10 μM of the PI3′K/AKT inhibitor; LY294002 (hereafter LY) or the ERK inhibitor; PD98059 (hereafter PD) for 24 h. Surprisingly, LY and PD beside inactivating AKT and ERK, respectively with no effect on the total proteins, both also significantly decreased BRCA1-IRIS expression (Figure 2H). In contrast, BRCA1-IRIS silencing or inactivation not only decreased BRCA1-IRIS expression level, but also total AKT and p-AKT levels in both cell lines (Figure 2H). Concurrently, LY, IRIS peptide and BRCA1-IRIS silencing all significantly decreased p-FOXO3a level and increased total FOXO3a level in both cell lines, which again was correlated with significant decrease in MDM2 and/or Skp2 expression (Figure 2H). Surprisingly, LY and IRIS peptide (and to a lesser degree BRCA1-IRIS silencing) had little effect on total ERK expression in this experiment but increased the level of p-ERK in both cell lines (Figure 2H). Taken together, these data, in addition to confirming the specificity of the peptide, they define a molecular mechanism of action for IRIS peptide in TNBC cell survival, a positive-feedback loop between BRCA1-IRIS and AKT signaling in both cell lines (see model in Figure 2I).

### BRCA1-IRIS overexpression induces in HME cells, while silencing or inactivation inhibits aggressiveness in TNBC cells, *in vitro*

**Figure 3:**
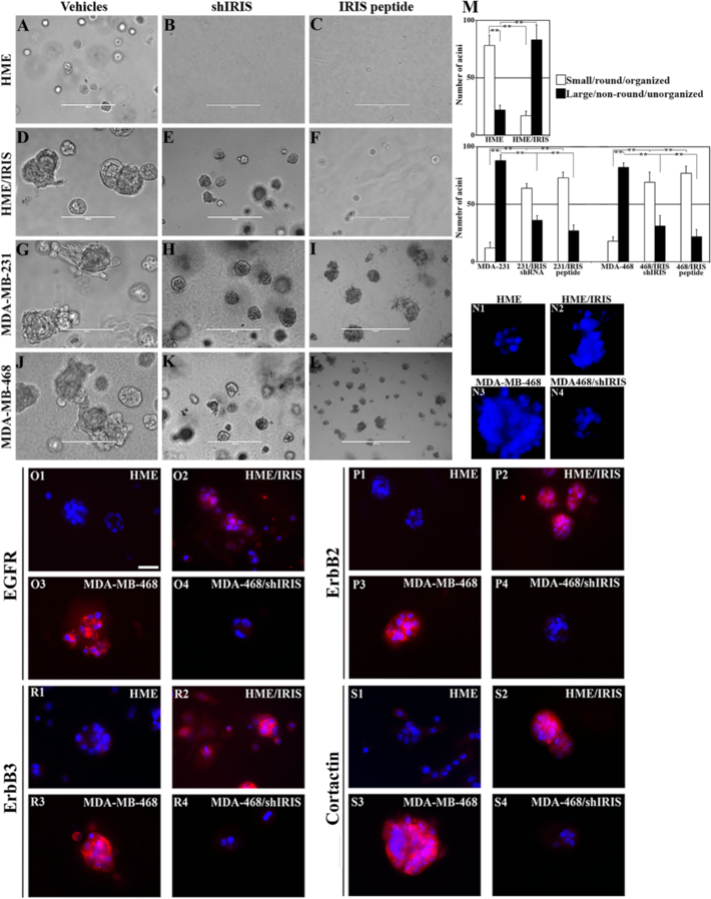
**BRCA1-IRIS overexpression promotes aggressiveness in HME cells, while inactivation inhibits it in TNBC cells.** Representative images showing acini formation in low growth factor matrigel-coated wells in the presence of vehicle, shIRIS or IRIS peptide in HME **(A**-**C)** HME/IRIS **(D-F)** MDA-MB-231 **(G-I)** and MDA-MB-468 **(J-L)** cell lines at day 10. IRIS peptide was added at 0.5 μM (HME) or 5 μM (the other cell lines). Scale bar in A-L = 400 μm. **(M)** Quantitative analysis of the number and phenotype of acini formed using HME and HME/IRIS (upper) or MDA-MB-231 and MDA-MB-468 (lower) following the above mentioned treatments at day 10. **(N1-N4)** Acini formed by HME, HME/IRIS, MDA-MB-468 and MDA-MB-468/shIRIS, respectively. The expression of the indicated proteins in acini formed using HME **(O1, P1, R1 and S1)**, HME/IRIS **(O2, P2, R2 and S2)**, MDA-MB-468 **(O3, P3, R3 and S3)** and MDA-MB-468/shIRIS **(O4, P4, R4 and S4)** cells. Scale bar in O1-S4 = 100 μm. HME, human mammary epithelial cells; TNBC, triple negative breast cancer.

Next, we asked whether BRCA1-IRIS depletion or inactivation impacts TNBC cells aggressiveness. To evaluate that, we used one of the best assays, ‘growth in three-dimensional culture’. HME, HME/IRIS, MDA-MB-231 or MDA-MB-468 cells stably expressing shcontrol or shIRIS were layered on matrigel-coated wells. Additionally, parental cell lines layered on matrigel-coated wells were grown in the presence of scrambled or IRIS peptide (added every third day). Ten days later, HME cells formed acini that were small/round/organized, composed of 40 to 50 cells and hollow in the middle (see Figure 3A and N1). In contrast, at day 10, acini formed by HME/IRIS (Figure 3D), MDA-MB-231 (Figure 3G) or MDA-MB-468 (Figure 3J) were much larger, non-round and unorganized with large protrusions, and were filled with cells on the inside resembling DCIS (Figure 3N2 and N3).

Moreover, BRCA1-IRIS depletion (Figure 3B) or inactivation (Figure 3C) completely abolished formation of acini by HME cells and converted HME/IRIS (Figure 3E and F, respectively), MDA-MB-231 (Figure 3H and I, respectively) and MDA-MB468 (Figure 3K and L, respectively) acini into smaller/rounder/organized acini. These acini also lacked protrusions and most importantly were hollow on the inside (see for example Figure 3N4 for MDA-MB-468 silenced cells). Quantitatively, BRCA1-IRIS overexpression led to more than threefold increase in HME cells aggressiveness (defined as generation of non-round, non-organized, large filled with cells acini, Figure 3M, upper), whereas depletion or inactivation reduced that phenotype in TNBC cells by more than threefold (defined as generation of round, organized, small and hallow acini Figure 3M, lower).

### BRCA1-IRIS overexpression induces in HME cells, while silencing inhibits aggressive biomarkers expression in TNBC cells, *in vitro*

To correlate these data with expression of aggressiveness biomarkers, similar acini were processed for immunofluorescence (IF) labeling with EGFR, ErbB2, ErbB3 and cortactin antibodies. HME acini showed low expression levels of all biomarkers that when found were, as expected, restricted to the apical side of the acini (see Figure 3O1, P1, R1 and S1). In contrast, HME/IRIS acini showed increase expression of all biomarkers, which were expressed at the apical as well as the basolateral side of the acini (Figure 3O2, P2, R2 and S2). MDA-MB-468 acini showed high levels and unrestricted expression of all aggressiveness biomarkers (Figure 3O3, P3, R3 and S3), whereas BRCA1-IRIS silencing in MDA-MB-468 cells not only decreased the expression of all aggressiveness biomarkers, but also restricted it to the apical side (Figure 3O4, P4, R4 and S4). Taken together, these data suggest that BRCA1-IRIS overexpression triggers a polarization, epithelial to mesenchymal transition (EMT) and aggressiveness in HME cells, which could explain the invasive, aggressiveness and unrestricted growth of HME/IRIS cells in three-dimensional matrigel and SCID mice [38], and that BRCA1-IRIS silencing in TNBC cells reverse these phenotypes.

### BRCA1-IRIS silencing or inactivation inhibits tumor-initiating phenotype in TNBC cells, *in vitro*

**Figure 4:**
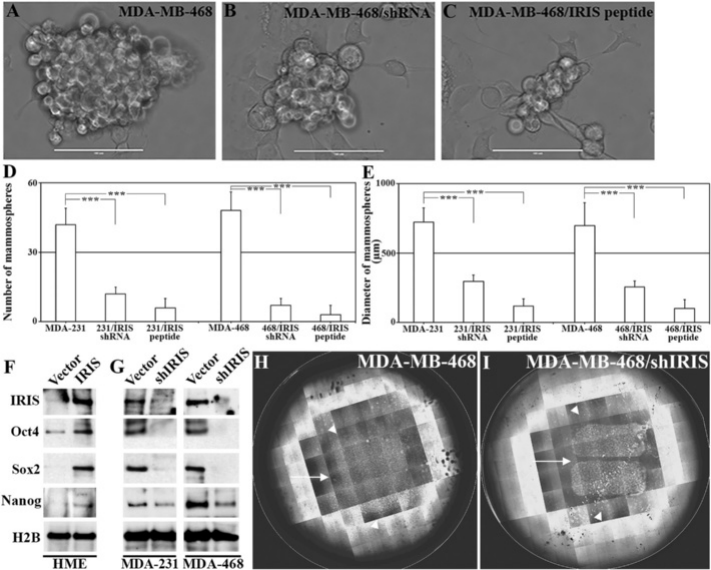
**BRCA1-IRIS overexpression promotes tumor-initiating phenotype in HME cells, while inactivation suppresses it in TNBC cells. (A-C)** Representative images showing mammosphere formation in MDA-MB-468 cells following control treatments **(A)**, BRCA1-IRIS silencing **(B)** or inactivation using IRIS peptide **(C)** at day 10. Scale bar in A-C = 1,000 μm. Quantitative analysis of the number **(E)** or diameter **(F)** of mammospheres developed using MDA-MB-231 or MDA-Mb-468 cells after vehicles, BRCA1-IRIS silencing or BRCA1-IRIS inactivation using IRIS peptide. **(G)** The expression of the indicted stemness biomarkers in HME or BRCA1-IRIS overexpressing HME cells or MDA-MB-231 and MDA-MB468 expressing or silenced from BRCA1-IRIS. Representative images of the migration of MDA-MB-468 **(H)** or MDA-MB-468 expressing IRIS shRNA **(I)**. In both images arrows show intervening spaces left by the insert that were filled by MDA-MB-468 (24 h later) and not in BRCA1-IRIS-silenced MD-MB-468 and in both images arrowheads show the distance MDA-MB-468 cells travelled outward and the lack of such migration in BRCA1-IRIS-silenced MDA-MB-468 cells. HME, human mammary epithelial cells; TNBC, triple negative breast cancer.

It has been suggested recently that a population with tumor-initiating (that is breast cancer stem-like) capabilities exists within TNBC tumors. To evaluate whether BRCA1-IRIS inactivation suppress stemness phenotype in TNBC cells, MDA-MB-231 and MDA-MB-468 cells stably expressing shcontrol or shIRIS were plated in ultra-low binding dishes. Additionally parental cells were also plated in ultra-low binding dishes and treated with scrambled or IRIS peptide readded to cells every third day. Mammospheres - a hallmark of breast cancer stem-like cells - formed within 10 days were then counted and photographed. Control shRNA expressing or scrambled peptide-treated MDA-MB-231 or MDA-MB-468 cells formed many large-size mammospheres (Figure 4A, for MDA-MB-468, identical result was documented for MDA-MB-231). BRCA1-IRIS silenced (Figure 4B, for MDA-MB-468, identical data was documented for MDA-MB-231) or inactivated (Figure 4C, for MDA-MB-468, identical data was documented for MDA-MB-231) showed much smaller mammospheres. Quantitatively, BRCA1-IRIS silencing or inactivation reduced the ability of both cell lines to form mammospheres by approximately threefold (Figure 4D) and whenever formed their size was approximately threefold smaller than control cells (Figure 4E).

### BRCA1-IRIS induces in HME cells, whereas silencing inhibits stemness biomarkers expression in TNBC cells, *in vitro*

To investigate whether altering BRCA1-IRIS alters stemness biomarkers expression, HME cells were induced to overexpress BRCA1-IRIS or TNBC cells were silenced from BRCA1-IRIS, and expression of stemness biomarkers, Oct-4, Sox-2 and Nanog was analyzed. BRCA1-IRIS overexpression significantly increased expression of these biomarkers in HME cells (Figure 4F). Conversely, BRCA1-IRIS silencing significantly decreased the expression of these biomarkers in MDA-MB-231 and MDA-MB-468 cells (Figure 4G). Taken together, these data suggest that inactivating BRCA1-IRIS suppresses the tumor-initiating phenotype, implicated in aggressiveness and recurrence of TNBC cells.

### BRCA1-IRIS silencing or inactivation inhibits migration and invasion in TNBC cells, *in vitro*

TNBC tumor cells often possess enhanced migration and invasion capabilities. To assess whether BRCA1-IRIS plays a role in these phenotypes as well, we plated MDA-MB-468 cells stably expressing shcontrol or shIRIS inside inserts placed in the middle of wells of 6-well plates. When confluent, inserts were removed, dying and dislodged cells were washed away and cells were allowed to migrate for 24 h. MDA-MB-468 cells expressing shcontrol showed robust migration capabilities as evident by filling the empty space left after the removal of the insert (see white arrow in Figure 4H) as well as the outward migration (see arrowheads in Figure 4H). BRCA1-IRIS-silenced MDA-MB-468 cells, on the other hand, almost completely lost their migratory ability (see white arrows and arrowheads in Figure 4I).

**Figure 5:**
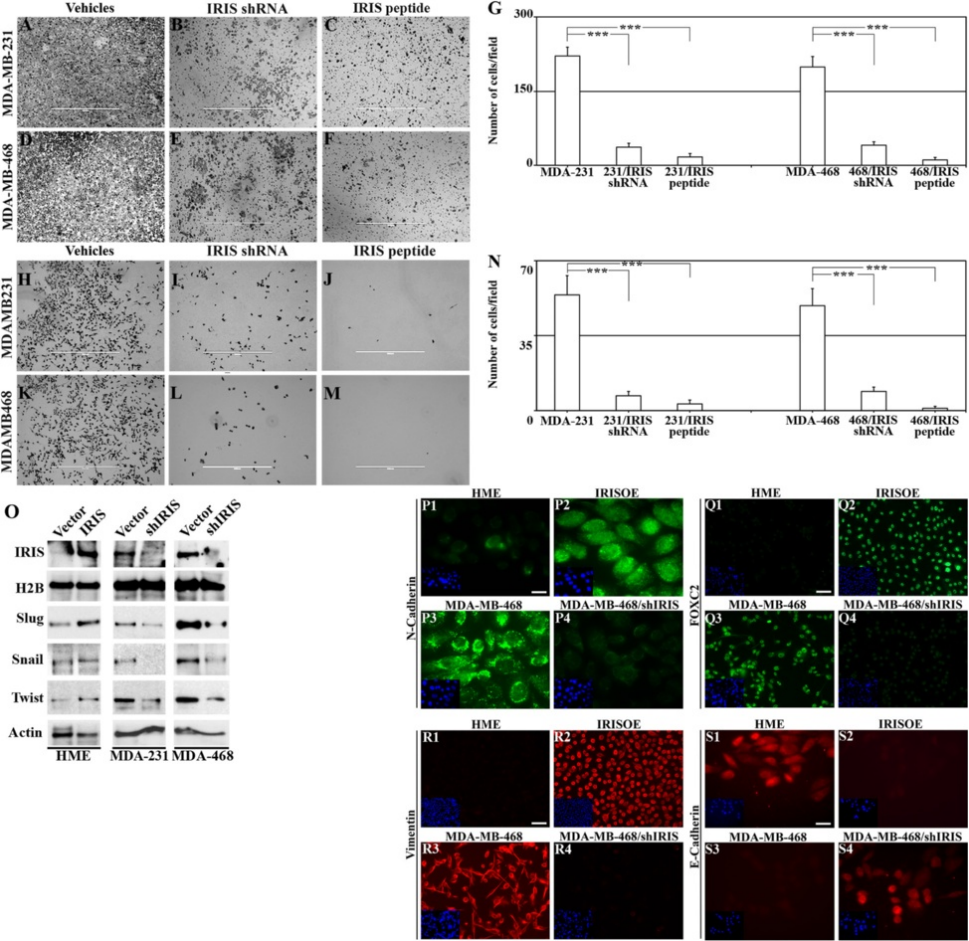
**BRCA1-IRIS overexpression promotes EMT and invasion in HME cells, while inactivation suppresses them in TNBC cells. (A)** Representative images showing the invasion ability of MDA-MB-231 **(A** and **H)** and MDA-MB-468 **(D** and **K)** cells through matrigel-coated Boyden chambers and the significant retardation of this invasion ability following BRCA1-IRIS silencing in MDA-MB-231 **(B** and **I)** and MDA-MB-468 **(E** and **L)** or BRCA1-IRIS inactivation using IRIS peptide in MDA-MB-231 **(C** and **J)** and MDA-MB-468 **(F** and **M)** cells on day 7. Scale bars in A-F and H-M = 1,000 μm. Quantitative analysis of invasive ability of the indicated cells shown as cells travelled to the other side of the transwells **(G)** or jumped to the lower well of the Boyden chamber **(N). (O)** The expression of the indicated EMT biomarkers in HME or BRCA1-IRIS overexpressing HME cells as well as MDA-MB-231 or MDA-MB-468 cells expressing or silenced from BRCA1-IRIS. Please note that BRCA1-IRIS and H2B blots used are the same as those used in Figure 4G. The expression of the indicated EMT/invasion biomarkers in HME **(P1, Q1, R1, and S1)**, BRCA1-IRIS-overexpressing HME **(P2, Q2, R2 and S2)**, MDA-MB-468 **(P3, Q3, R3 and S3)** or MDA-MB-468 silenced from BRCA1-IRIS **(P4, Q4, R4 and S4)** cells. Scale bars in P1-P4 and S1-S4 = 20 μm, and in Q1-Q4 and R1-R4 = 50 μm. EMT, epithelial to mesenchymal transition; HME, human mammary epithelial cells; TNBC, triple negative breast cancer.

To study whether BRCA1-IRIS silencing or inactivation affects TNBC cells invasion ability, MDA-MB-231 and MDA-MB-468 cells expressing shcontrol or shIRIS were layered on matrigel-coated Boyden chambers transwells. Additionally, parental cells were layered on transwells and grown in the presence of scrambled or IRIS peptide. To migrate to the lower side of the chamber or the bottom well, cells must first digest the matrigel. Four days later, a significant number of control cells (expressing empty vector or treated with scrambled peptide) invaded the matrigel and migrated to the lower side of the chambers (Figure 5A for MDA-MB-231, 5D for MDA-MB-468) and the bottom well (Figure 5H for MDA-MB-231, 5K for MDA-MB-468) in both cell lines. BRCA1-IRIS-silenced (Figure 5B and I, for MDA-MB-231, 5E and L for MDA-MB-468) or -inactivated (Figure 5C and J for MDA-MB-231, 5F and M for MDA-MB-468) cells almost completely lost their ability to invade and migrate. Quantitatively, BRCA1-IRIS silencing or inactivation reduced the invasive/migratory ability of these TNBC cells by approximately 100-fold (see Figure 5G for cells on the reverse side of the transwell, and Figure 5N for cells on the bottom well).

### BRCA1-IRIS overexpression induces in HME cells, whereas silencing inhibits EMT and invasion biomarkers expression in TNBC cells, *in vitro*

To investigate whether altering BRCA1-IRIS alters EMT and invasion biomarkers expression, HME cells were induced to overexpress BRCA1-IRIS or TNBC cells were silenced from BRCA1-IRIS. BRCA1-IRIS overexpression in HME cells significantly enhanced expression of EMT biomarkers, such as slug, snail, and twist (Figure 5O), as well as invasion biomarkers, such as N-cadherin (compare Figure 5P2 to P1), FOXC2 (compare Figure 5Q2 to Q1) and vimentin (compare Figure 5R2 to R1) and suppressed expression of E-cadherin (compare Figure 5S2 to S1). Conversely, BRCA1-IRIS silencing in MDA-MB-231 or MDA-MB-468 cells significantly reduced expression of slug, snail and twist (Figure 5O), as well as N-cadherin (compare Figure 5P4 to P3), FOXC2 (compare Figure 5Q4 to Q3) and vimentin (compare Figure 5R4 to R3) and enhanced E-cadherin expression (compare Figure 5S4 to S3). Taken together, these data suggest that BRCA1-IRIS overexpression triggers EMT, migration and invasion in TNBC cells.

### Elevated BRCA1-IRIS and survivin, while lack of FOXO3a expression in aggressive human breast tumors

**Figure 6:**
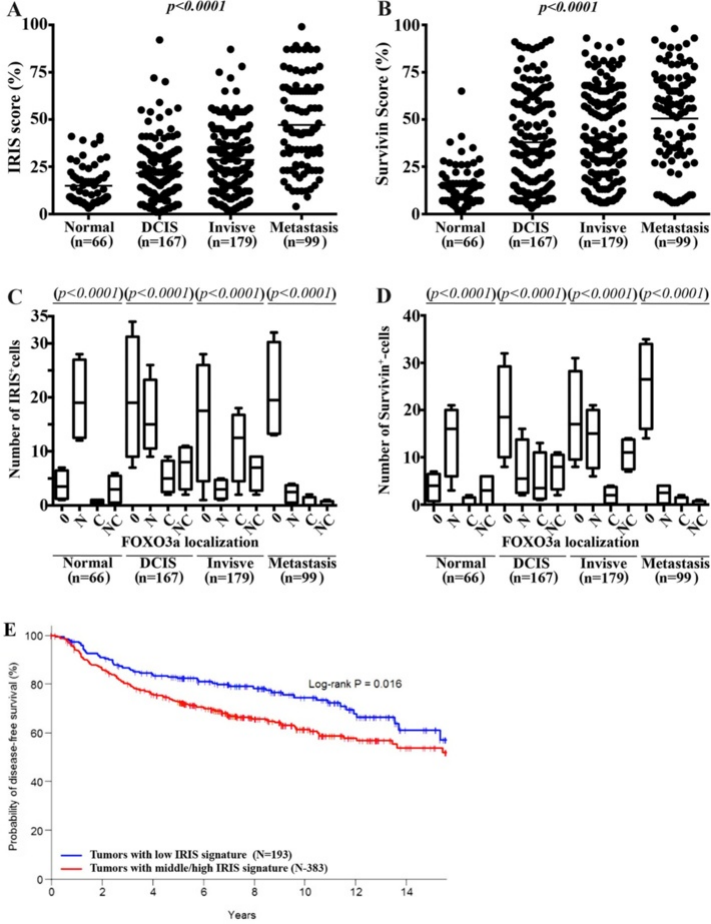
**Elevated BRCA1-IRIS and survivin and lack of FOXO3a expression correlates with breast tumors aggressiveness.** Paraffin-embedded tissue microarray sections were examined by immunohistochemistry with anti-BRCA1-IRIS, survivin and FOXO3a mAb. **(A** and **B)** BRCA1-IRIS and survivin, respectively staining scores in normal (n = 66), DCIS (n = 167), invasive (n = 179) and metastatic (n = 99) breast cancer tissue samples. **(C** and **D)** BRCA1-IRIS and survivin, respectively staining scores per field as compared to the localization of FOXO3a in each cell in normal (n = 66), DCIS (n = 167), invasive (n = 179) and metastatic (n = 99) breast cancer tissue samples. **(E)** Percentage of probability of disease-free survival in patients with TNBC tumors overexpressing low (blue line) vs. middle/high (red line) levels of EGFR/AKT/MDM2/Skp2/survivin as a surrogate for BRCA-IRIS overexpression. DCIS, ductal carcinoma *in situ*; FOXO3a, Forkhead box class OAKT3a; mAb, monoclonal antibody; TNBC, triple negative breast cancer.

Previously, we showed elevated BRCA1-IRIS expression in approximately 80% of breast tumors (>800 tumor samples were analyzed) that was correlated with elevated p-AKT and survivin expression (Ref. [38]). To correlate these data to FOXO3a expression, tissue microarrays consisted of normal/cancer adjacent (n = 66), DCIS (n = 167), invasive (n = 179) and metastatic (n = 99) samples were immunohistochemically stained with anti-BRCA1-IRIS, -survivin and -FOXO3a antibodies. Semi-quantitative scoring analysis (see Methods) showed that BRCA1-IRIS (*P* <0.0001, Figure 6A) and survivin (*P* <0.0001, Figure 6B) expression significantly increased concurrently with the increase in tumor aggressiveness. Indeed, aside from the fact that more cells per field showed upregulation of BRCA1-IRIS and survivin, the intensity of the staining per cell also increased as disease progressed (data not shown).

Since p-AKT phosphorylation restricts FOXO3a nuclear translocation and promotes its degradation, we evaluated the presence as well as the cellular localization of FOXO3a in these tumors. We evaluate the nuclear (N), cytoplasmic (C), both (NC) or negative (0) expression of FOXO3a. We found that FOXO3a staining was N in the majority of BRCA1-IRIS expressing (IRIS^+^) and survivin-expressing (survivin^+^) normal tissues (all *P* <0.0001*,* Figure 6C and D). In contrast, the majority of IRIS^+^ or survivin^+^ DCIS tumors showed 0, with few tumors showing N staining (*P* <0.0001, Figure 6C and D). The majority of IRIS^+^ invasive tumors showed 0, with few tumors showing C FOXO3a staining, whereas the majority of the survivin^+^ invasive tumors were 0, with few tumors showing N FOXO3a staining (*P* <0.0001, Figure 6C and D). Finally, nearly all IRIS^+^ or survivin^+^ metastatic breast cancer tissues were 0 for FOXO3a (*P* <0.0001, Figure 6C and D). Taken together, these data suggest that during breast cancer progression, elevated BRCA1-IRIS expression activates AKT that decreases FOXO3a expression leading to elevated survivin expression.

### Low disease-free survival in patients with elevated BRCA1-IRIS signature-expressing TNBC tumors

Based on the data presented above we concluded that BRCA1-IRIS-overexpressing TNBC cells show concurrent elevation in the expression of ‘EGFR, AKT, MDM2, Skp2 and survivin’. Considering this short list as a BRCA1-IRIS-overexpression signature in TNBC tumors, we conducted an association assessment of this signature and disease-free survival (DFS) using a combined breast cancer cohort from seven Gene Expression Omnibus (GEO) studies (GSE2034, GSE2603, GSE3494, GSE4922, GSE6532, GSE7390 and GSE12093). Patients with positive status for estrogen, progesterone and HER2 were excluded leading to a size of 576 cases. The standardized BRCA1-IRIS signature expression levels were pooled together and considered as explanatory variables in a Cox model on DFS. Based on the estimation result of the Cox model, we developed a prognostic index combining the expression levels of BRCA1-IRIS overexpression signature: ‘Prognostic index = EGFR + 2 x AKT + 2 x MDM2 + 2 x Skp2 + 2 x survivin*’*, which was evaluated for the entire sample. The patient cohort was further categorized into low- versus middle/high-expressing groups by using the lower tertile as the cutoff value. Using Kaplan-Meier method and group-wise comparison in DFS done using the log-rank test, this analysis showed that among the cohort tested, patients with middle/high expression levels of BRCA1-IRIS overexpression signature showed significantly (Log-rank *P* = 0.016) shorter DFS compared to those showing low expression levels (Figure 6E). Taken together, these data suggest that elevated BRCA1-IRIS level is a poor overall prognosis in patients with TNBC disease.

### BRCA1-IRIS silencing or inactivation blocks TNBC tumor formation, maintenance and sensitizes them to low paclitaxel concentrations, *in vivo*

**Figure 7:**
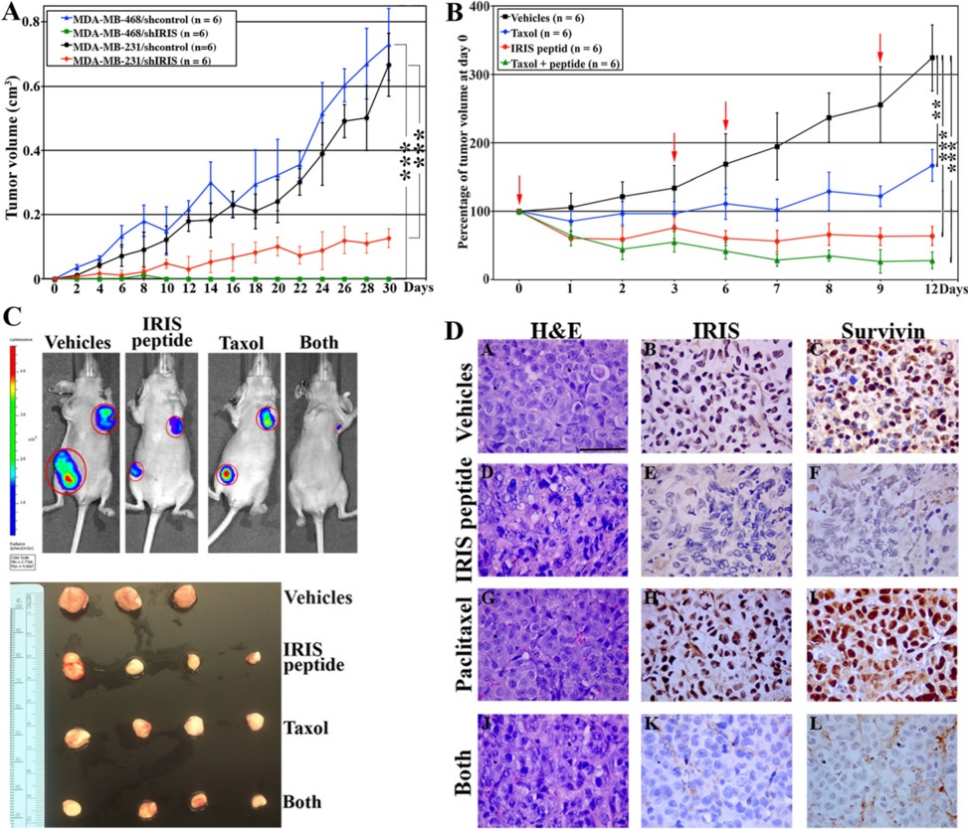
**BRCA1-IRIS overexpression promotes TNBC formation and maintenance, while inactivation sensitizes them to low paclitaxel concentrations. (A)** Volumes of tumors developed in SCID mice using MDA-MB-231/shcontrol (black line, n = 6), MDA-MB-231/shIRIS (red line, n = 6), MDA-MB-468/shcontrol (blue line, n = 6) or MDA-MB-468/shIRIS (green line, n = 6). **(B)** The effect of vehicle (black line, n = 6), paclitaxel (10 mg/kg, delivered i.p., blue line, n = 6), IRIS peptide (10 mg/kg, delivered i.t., red line, n = 6), or both (at half the concentrations, delivered through the same routes, green line, n = 6) on an established MDA-MB-468 tumors. Red arrows show the times of the drug administration. ^**^ = *P* ≤0.001 and ^***^ = *P* ≤0.0001. **(C)** Shows representative images of treated mice as described in **(B)** at day 12 (upper) or representative images of tumors isolated from these mice following the treatments also at day 12 (lower). **(D)** Representative images of the sections from tumors shown in **(B** and **C)** stained with H&E (left), BRCA1-IRIS (middle), survivin (right) antibodies. Scale bar is D = 100 μm. H&E, hematoxylin and eosin; i.p., intraperitoneally; i.t., intratumorally; TNBC, triple negative breast cancer.

We showed previously that BRCA1-IRIS-overexpressing HME cells form aggressive TNBC tumors lacking BRCA1 expression [38]. To evaluate whether BRCA1-IRIS overexpression is indeed required for TNBC tumor formation and/or maintenance, MDA-MB-231 or MDA-MB-468 (2 x 10^6^) cells expressing shcontrol or shIRIS were injected in mammary fat pads (second left and fourth right) of 6- to 8-week-old female SCID mice (mice, n = 6/cell line → tumors, n = 12/cell line). Control shRNA-expressing cells formed tumors that reached approximately 750 mm^3^ within 4 weeks (black line for MDA-MB-231 and blue line for MDA-MB-468 in Figure 7A). BRCA1-IRIS-silenced MDA-MB-231 formed very small tumors that were approximately 100 mm^3^ by 4 weeks (red line in Figure 7A), and BRCA1-IRIS-silenced MDA-MB-468 cells failed completely to form any tumors (green line in Figure 7A). These data, suggest that BRCA1-IRIS overexpression is required for TNBC tumor formation.

Moreover, MDA-MB-468 or MDA-MB-231 (2 x 10^6^) cells were injected in mammary fat pads (second left and fourth right) of 6- to 8-week-old Nu/Nu female mice (mice, n = 24 → tumors, n = 48). When tumors reached approximately 0.1 cm^3^ in volume, the mice were divided into four groups that were treated with vehicles (that is DMSO injected intraperitoneally (i.p.) + scrambled peptide injected directly into tumors (i.t.), mice, n = 6 → tumors, n = 12), paclitaxel (10 mg/kg, i.p., mice, n = 6 → tumors, n = 12), IRIS peptide at (10 mg/kg, i.t., mice, n = 6 → tumors, n = 12), or paclitaxel + IRIS peptide (at half the concentrations, same routes, mice, n = 6 → tumors, n = 12). Drugs were delivered every third day for total of four injections (see red arrows in Figure 7B). Tumors were measured using caliper three times per week and collected at the end of the experiment (day 12). At day 12, tumors in vehicle-treated mice became approximately 3.5 times their original size at day 0 (see black line in Figure 7B and 7C). Impressively, at day 12, tumors in IRIS peptide-treated mice, actually shrunk to approximately 25% their original size at day 0 (see red line in Figure 7B and 7C). Taken together, these data suggest that BRCA1-IRIS overexpression is important for TNBC tumor maintenance.

Moreover, at day 12, paclitaxel treatment showed some effect, but tumors still grew to approximately 1.5 times their original size at day 0 (see blue line in Figure 7B and 7C). More importantly, tumors treated with the combination ‘IRIS peptide + paclitaxel’ (at only half the above concentrations) shrunk to <15% of their original size at day 0 (see green line in Figure 7B and 7C). Taken together, these data suggest that inactivating BRCA1-IRIS sensitizes TNBC tumors to low paclitaxel concentrations, *in vivo*.

### BRCA1-IRIS inactivation suppresses paclitaxel-induced overexpression in BRCA1-IRIS → survivin, *in vivo*

Finally, to correlate the above *in vivo* efficacy data of IRIS peptide to expression of BRCA1-IRIS and hence survivin in these tumors, we stained adjacent sections from the tumors developed above with hematoxylin and eosin (H&E) or with IHC for BRCA1-IRIS or survivin. As expected, in controls-treated tumors, BRCA1-IRIS and survivin expression was high (Figure 7D, upper row), and as described above, the expression increased even further by paclitaxel treatment (even though tumors were smaller, Figure 7D, third row). In contrast, the expression of both proteins was completely abolished in tumors treated with IRIS peptide (Figure 7D, second row) or the combination (even at only half the concentrations, Figure 7D, fourth row). Taken together, these data could explain the acquired paclitaxel resistant recurrences observed in TNBC patients.

## Discussion

Development of chemotherapy-resistant recurrences plays a major role in breast cancer mortalities [43]. Paclitaxel promotes apoptosis in tumor cells by inducing a permanent mitotic arrest. However, adaptation that develops in some paclitaxel-treated tumor cells can lead to tumor progression [45,46]. BRCA1-IRIS expression is elevated in the majority of breast cancers, including TNBCs [38]. BRCA1-IRIS-overexpressing tumors show adverse outcomes, progression and metastasis [38].

Here, we present extensive evidence showing a paclitaxel-resistance-promoting role for BRCA1-IRIS overexpression in TNBC tumor cells, *in vitro* and *in vivo*. BRCA1-IRIS overexpression drastically diminishes paclitaxel efficacy as evidenced by decreased apoptosis of treated cells *in vitro* (Figure 2) and *in vivo* (Figure 7). Only following BRCA1-IRIS silencing or inactivation (using a novel BRCA1-IRIS inhibitory peptide) was the efficacy of paclitaxel restored. BRCA1-IRIS mediates this resistance by upregulating expression of survivin through activation of AKT and/or inactivation of FOXO3a *in vitro* and *in vivo*. In addition, increased expression of other prominent apoptosis-suppressing proteins, such as Bcl-2, Bcl-xL and NF-κB by BRCA1-IRIS could also play a role.

The most troubling observation in the studies described above is the fact that at low concentration, paclitaxel upregulates BRCA1-IRIS expression in normal (see above) as well as low BRCA1-IRIS-expressing breast cancer cells (not shown). This observation may suggest that paclitaxel promotes its own resistance in patients by selecting certain tumor cells to survive and repopulate the tumor by upregulating BRCA1-IRIS in them. More seriously still is the prospective that the generation of new tumor not just recurrent tumor developed after treatment from normal cells. The mechanism responsible for paclitaxel (low concentration)-induced BRCA1-IRIS expression is being investigated. However, overall, these findings indicate that BRCA1-IRIS upregulation is involved in TNBCs intrinsic and acquired paclitaxel resistance and its inhibition can be pursued as a therapeutic option to reverse this resistance in TNBC patients.

The specificity of BRCA1-IRIS-overexpression-induced acquired paclitaxel resistance is shown here by genetic manipulation of BRCA1-IRIS in three aggressive TNBC lines. In addition, we achieved sensitization to lower concentrations of paclitaxel-induced apoptosis, *in vitro* and *in vivo* and corresponding reduction in the aforementioned pathways when BRCA1-IRIS activity was reduced in these cell lines using the novel IRIS peptide. This was further supported by the fact that one of the most prominent effects of low paclitaxel concentration-induced resistance in HME cells was BRCA1-IRIS overexpression, which was followed by upregulation of the survival pathways described above. Taken together, these data strongly support the notion that whether intrinsically or acquired following paclitaxel (especially low concentration) treatment, the upregulation in BRCA1-IRIS in TNBC cells is a major obstacle against obtaining major efficacy for paclitaxel, especially in patients with metastatic breast cancer. We therefore propose that inhibiting BRCA1-IRIS expression and/or activity could sensitize these tumors to paclitaxel and perhaps as our data suggest, lower and less toxic concentrations of this chemotherapy.

Our data, especially with the IRIS peptide, seem to suggest that intact AKT is more important for TNBC than intact ERK pathway since prior to promoting cell death in TNBC cells, BRCA1-IRIS silencing or inactivation inactivated the AKT pathway but had no or the opposite effect on ERK pathway. However, we cannot rule out the possibility that to completely eradicate BRCA1-IRIS-overexpressing TNBC cells, ERK1/2 inhibitors must be combined with AKT and/or BRCA1-IRIS inhibitors. It is also possible - since we cannot distinguish between ERK1 or ERK2 activation in these assays - that the two act in a different manner. Interestingly, AKT - and to a lesser extent ERK - inactivation significantly decreased BRCA1-IRIS level in the TNBCs cell lines tested. This implies a feed-forward mechanism is at work in TNBCs (possibly other cell types as well). It is possible that p-AKT enhances BRCA1-IRIS expression, which enhances AKT expression and activation. One of two possibilities might account for this phenomenon. The first is that through silencing BRCA1 (see [38,44]), BRCA1-IRIS is able to prevent AKT ubiquitination and degradation as was previously shown. Alternatively, the two events could be unconnected and merely a consequence of other activities in TNBC cells. Whatever the explanation is, a positive feedback mechanism between BRCA1-IRIS and AKT pathways is directly correlated with the BRCA1-IRIS chemotherapy resistance-inducing role in TNBC survival.

Mechanistically, BRCA1-IRIS-dependent paclitaxel resistance could be mediated by pro-survival autocrine signaling loops, such as those shown here, namely EGF/EGFR-ErbB2 and NRG1/ErbB2-ErbB3. Although paclitaxel-mediated increases in the expression of some oncogenes have been previously reported in both human patients with breast cancer [46] and experimental breast cancer models [47], this is the first study that analyzed co-expression of BRCA1-IRIS and ErbB family members. This evidence strongly suggests that therapy-induced activation of BRCA1-IRIS pathway promotes tumor cell survival through autocrine signaling loops. However, secondary pathways initiated from tumor stromal cells are also possible [48]. Indeed, the fact that intrinsic or paclitaxel-acquired upregulation of BRCA1-IRIS induced expression and activation of NF-κB, as evidenced by increased expression and nuclear accumulation of p65 [44], could lead to, among other effects, transcription and secretion of a plethora of inflammatory cytokines, such as interleukin 6 (IL-6), interleukin 8 (IL-8), tumor necrosis factor alpha (TNF-α) and monocyte chemotactic protein 1 (MCP-1) that alter the tumor microenvironment through autocrine and paracrine loops [49]. Many of these cytokines were recently shown to act in autocrine but mostly in paracrine fashion between tumor cells and the surrounding microenvironment. These factors bind on the surface of stromal cells to specific receptors and induce expression of other factors that promote breast cancer cells aggressiveness, in this case in a paracrine manner [50].

Interestingly, our data also present a novel yet expected conclusion [51], which suggest that BRCA1-IRIS overexpression, which has been shown earlier to be associated with metastasis and poor survival in invasive ductal breast carcinoma is linked to uncoupling of the AKT-FOXO3a signaling axis. This conclusion has been reached based on the lack of FOXO3a in the nucleus in more aggressive tumors, which is known to overexpress BRCA1-IRIS and activated AKT. Thus it was predicted that survivin expression would be positively correlated with BRCA1-IRIS overexpression in aggressive and drug-resistant tumors. This also can explain the fact that paclitaxel failed to induce cell cycle arrest in BRCA1-IRIS-overexpressing cells, except after BRCA1-IRIS silencing or inactivation since this would require FOXO3a nuclear translocation to activate gene expression of cell cycle inhibitors, such as p21 and p27, both were not expressed in BRCA1-IRIS-overexpressing cells with intrinsically or acquired paclitaxel resistance.

## Conclusions

BRCA1-IRIS signaling in TNBC cells may significantly reduce chemotherapeutic efficacy by promoting survival of damaged cells, which is most likely a major reason for the prevalent metastasis detected in TNBC patients overexpressing BRCA1-IRIS [38]. BRCA1-IRIS inactivation could be pursued as first-line therapy to combat TNBC (as well as other subtypes) formation, progression and their drug-resistant recurrence in order to reduce TNBC-related mortalities.

## Abbreviations

DCIS: ductal carcinoma *in situ*
DFS: disease-free survival
EGF: epidermal growth factor, NRG1, neurogulin 1
EGFR: ErbB2 and ErbB3, epidermal growth factor receptor 1, 2 and 3, respectively
EMT: epithelial to mesenchymal transition
ER: estrogen receptor
FBS: fetal bovine serum
FOXO3a: Forkhead box class OAKT3a
H&E: hematoxylin and eosin
HER2: human epidermal growth factor receptor 2
HME: human mammary epithelial cells
HOSE: human ovarian surface epithelial cells
IAP: inhibitor of apoptosis protein
IF: immunofluorescence
IHC: immunohistochemistry
IL-6: interleukin 6
IL-8: interleukin 8
i.p.: intraperitoneally
i.t.: intratumorally
MAPK: mitogen-activated protein kinases
MCP1: monocyte chemotactic protein 1
NF-κB: nuclear factor kappa B
PR: progesterone receptor
PKB/AKT: protein kinase B
shRNA: small hairpin RNA
siRNA: small interfering RNA
TERT: telomerase reverse transcriptase
TNBC: triple negative breast cancer
TNF-α: tumor necrosis factor alpha

## References

[1] Ozols RF (2000). Paclitaxel (Taxol)/carboplatin combination chemotherapy in the treatment of advanced ovarian cancer. Semin Oncol..

[2] Kosmas C, Tsavaris NB, Polyzos A, Kalofonos HP, Sepsas E, Malamos NA (2000). A phase II study of paclitaxel-ifosfamide-cisplatin combination in advanced non-small cell lung carcinoma. Cancer..

[3] De Lena M, Latorre A, Calabrese P, Catino A, Lorusso V, Mazzei A (2000). High efficacy of paclitaxel and doxorubicin as first-line therapy in advanced breast cancer: a phase I-II study. J Chemother..

[4] Mekhail TM, Markman M (2002). Paclitaxel in cancer therapy. Expert Opin Pharmacother..

[5] Jiménez B, Trigo JM, Pajares BI, Sáez MI, Quero C, Navarro V (2013). Efficacy and safety of weekly paclitaxel combined with cetuximab in the treatment of pretreated recurrent/metastatic head and neck cancer patients. Oral Oncol..

[6] Wong YN, Litwin S, Vaughn D, Cohen S, Plimack ER, Lee J (2012). Phase II trial of cetuximab with or without paclitaxel in patients with advanced urothelial tract carcinoma. J Clin Oncol..

[7] Pal SK, Yamzon J, Sun V, Carmichael C, Saikia J, Ferrell B (2013). Paclitaxel-based high-dose chemotherapy with autologous stem cell rescue for relapsed germ cell tumor: clinical outcome and quality of life in long-term survivors. Clin Genitourin Cancer..

[8] Dhillon T, Stebbing J, Bower M (2005). Paclitaxel for AIDS-associated Kaposi’s sarcoma. Expert Rev Anticancer Ther..

[9] Westerhoff HV, Riethorst A, Jongsma AP (2000). Relating multidrug resistance phenotypes to the kinetic properties of their drug-efflux pumps. Eur J Biochem..

[10] Kadoyama K, Kuwahara A, Yamamori M, Brown JB, Sakaeda T, Okuno Y (2011). Hypersensitivity reactions to anticancer agents: data mining of the public version of the FDA adverse event reporting system. AERS. J Exp Clin Cancer Res..

[11] Tanimukai H, Kanayama D, Omi T, Takeda M, Kudo T (2013). Paclitaxel induces neurotoxicity through endoplasmic reticulum stress. Biochem Biophys Res Commun..

[12] Symmans FW (2001). Breast cancer response to paclitaxel in vivo. Drug Resist Updat..

[13] Zaffaroni N, Daidone MG (2002). Survivin expression and resistance to anticancer treatments: perspectives for new therapeutic interventions. Drug Resist Updat..

[14] Nassar A, Lawson D, Cotsonis G, Cohen C (2008). Survivin and caspase-3 expression in breast cancer: correlation with prognostic parameters, proliferation, angiogenesis, and outcome. Appl Immunohistochem Mol Morphol..

[15] Tang C, Lu YH, Xie JH, Wang F, Zou JN, Yang JS (2009). Downregulation of survivin and activation of caspase-3 through the PI3K/Akt pathway in ursolic acid-induced HepG2 cell apoptosis. Anticancer Drugs..

[16] Su L, Wang Y, Xiao M, Lin Y, Yu L (2010). Up-regulation of survivin in oral squamous cell carcinoma correlates with poor prognosis and chemoresistance. Oral Surg Oral Med Oral Pathol Oral Radiol Endod..

[17] Xie YL, An L, Jiang H, Wang J (2012). Nuclear survivin expression is associated with a poor prognosis in Caucasian non-small cell lung cancer patients. Clin Chim Acta..

[18] Waligórska-Stachura J, Jankowska A, Waśko R, Liebert W, Biczysko M, Czarnywojtek A (2012). Survivin–prognostic tumor biomarker in human neoplasms–review. Ginekol Pol..

[19] Erpolat OP, Gocun PU, Akmansu M, Karakus E, Akyol G (2012). High expression of nuclear survivin and Aurora B predicts poor overall survival in patients with head and neck squamous cell cancer. Strahlenther Onkol..

[20] Ekeblad S, Lejonklou MH, Stålberg P, Skogseid B (2012). Prognostic relevance of survivin in pancreatic endocrine tumors. World J Surg..

[21] Capalbo G, Rödel C, Stauber RH, Knauer SK, Bache M, Kappler M (2007). The role of survivin for radiation therapy. Prognostic and predictive factor and therapeutic target. Strahlenther Onkol.

[22] Pennati M, Folini M, Zaffaroni N (2007). Targeting survivin in cancer therapy: fulfilled promises and open questions. Carcinogenesis..

[23] Yang JY, Hung MC (2009). A new fork for clinical application: targeting forkhead transcription factors in cancer. Clin Cancer Res..

[24] Hagenbuchner J, Ausserlechner MJ (2013). Mitochondria and FOXO3: breath or die. Front Physiol..

[25] Hu MC, Lee DF, Xia W, Golfman LS, Ou-Yang F, Yang JY (2004). IkappaB kinase promotes tumorigenesis through inhibition of forkhead FOXO3a. Cell..

[26] Finnberg N, El-Deiry WS (2004). Activating FOXO3a, NF-kappaB and p53 by targeting IKKs: an effective multi-faceted targeting of the tumor-cell phenotype?. Cancer Biol Ther..

[27] Zhang Y, Gan B, Liu D, Paik JH (2011). FoxO family members in cancer. Cancer Biol Ther..

[28] Huang H, Tindall DJ (2011). Regulation of FOXO protein stability via ubiquitination and proteasome degradation. Biochim Biophys Acta..

[29] Sunters A (2003). Fernández de Mattos S, Stahl M, Brosens JJ, Zoumpoulidou G, et al. FoxO3a transcriptional regulation of Bim controls apoptosis in paclitaxel-treated breast cancer cell lines. J Biol Chem..

[30] Kikuchi S, Nagai T, Kunitama M, Kirito K, Ozawa K, Komatsu N (2007). Active FKHRL1 overcomes imatinib resistance in chronic myelogenous leukemia-derived cell lines via the production of tumor necrosis factor-related apoptosis-inducing ligand. Cancer Sci..

[31] Gomes AR, Brosens JJ, Lam EW (2008). Resist or die: FOXO transcription factors determine the cellular response to chemotherapy. Cell Cycle..

[32] ElShamy WM, Livingston D (2004). Identification of BRCA1-IRIS, a BRCA1 locus product. Nat Cell Biol..

[33] Furuta S, Jiang X, Gu B, Cheng E, Chen PL, Lee WH (2005). Depletion of BRCA1 impairs differentiation but enhances proliferation of mammary epithelial cells. Proc Natl Acad Sci U S A..

[34] Nakuci E, Mahner S, Direnzo J, ElShamy WM (2006). BRCA1-IRIS regulates cyclin D1 expression in breast cancer cells. Exp Cell Res..

[35] Hao L, ElShamy WM (2007). BRCA1-IRIS activates cyclin D1 expression in breast cancer cells by downregulating the JNK phosphatase DUSP3/VHR. Int J Cancer..

[36] Chock K, Allison JM, ElShamy WM (2010). BRCA1-IRIS overexpression abrogates UV-induced p38MAPK/p53 and promotes proliferation of damaged cells. Oncogene..

[37] Chock KL, Allison JM, Shimizu Y, ElShamy WM (2010). BRCA1-IRIS overexpression promotes cisplatin resistance in ovarian cancer cells. Cancer Res..

[38] Shimizu Y, Luk H, Horio D, Miron P, Griswold M, Iglehart D (2012). BRCA1-IRIS overexpression promotes formation of aggressive breast cancers. PLoS One..

[39] Integrated DNA Technologies. http://www.idtdna.com/Scitools/Applications/shRNA

[40] Grayson W, Cooper K (2003). Application of immunohistochemistry in the evaluation of neoplastic epithelial lesions of the uterine cervix and endometrium. Curr Diag Path..

[41] Hsu S-M, Raine L, Fanger H (1981). Use of avidin-biotin-peroxidase complex (ABC) in immunoperoxidase techniques: a comparison between ABC and unlabeled antibody (PAP) procedures. J Histochem Cytochem..

[42] Choudhury K, Yagle K, Swanson P, Krohn K, Rajendran J (2010). A robust automated measure of average antibody staining in immunohistochemistry images. J Histochem Cytochem..

[43] McCloskey DE, Kaufmann SH, Prestigiacomo LJ, Davidson NE (1996). Paclitaxel induces programmed cell death in MDA-MB-468 human breast cancer cells. Clin Cancer Res..

[44] Shimizu Y, Mullins N, Blanchard Z, ElShamy WM (2012). BRCA1/p220 loss triggers BRCA1-IRIS overexpression via mRNA stabilization in breast cancer cells. Oncotarget..

[45] Gerber B, Freund M, Reimer T (2010). Recurrent breast cancer: treatment strategies for maintaining and prolonging good quality of life. Dtsch Arztebl Int..

[46] Weaver B, Cleveland D (2005). Decoding the links between mitosis, cancer, and chemotherapy: the mitotic checkpoint, adaption, and cell death. Cancer Cell..

[47] Lee J, Swain S (2005). Development of novel chemotherapeutic agents to evade the mechanisms of multidrug resistance (MDR). Sem Oncol..

[48] Pusztai L, Mendoza T, Reuben J, Martinez M, Willey J, Lara J (2004). Changes in plasma levels of inflammatory cytokines in response to paclitaxel chemotherapy. Cytokine..

[49] Volk L, Flister M, Bivens C, Stutzman A, Desai N, Trieu V (2008). Nab-paclitaxel efficacy in the orthotopic model of human breast cancer is significantly enhanced by concurrent anti-vascular endothelial growth factor A therapy. Neoplasia..

[50] Eiró N, González L, González LO, Fernandez-Garcia B, Lamelas ML, Marín L (2012). Relationship between the inflammatory molecular profile of breast carcinomas and distant metastasis development. PLoS One..

[51] Chen J, Gomes AR, Monteiro LJ, Wong SY, Wu LH, Ng TT (2010). Constitutively nuclear FOXO3a localization predicts poor survival and promotes Akt phosphorylation in breast cancer. PLoS One..

